# Surgical Neurostimulation for Spinal Cord Injury

**DOI:** 10.3390/brainsci7020018

**Published:** 2017-02-10

**Authors:** Aswin Chari, Ian D. Hentall, Marios C. Papadopoulos, Erlick A. C. Pereira

**Affiliations:** 1 Academic Neurosurgery Unit, St George’s, University of London, London SW17 0RE, UK; aswinchari@gmail.com (A.C.); eacp@eacp.co.uk (E.A.C.P.); 2 Division of Brain Sciences, Faculty of Medicine, Imperial College London, London W6 8RF, UK; 3 The Miami Project to Cure Paralysis and Department of Neurological Surgery, Miller School of Medicine, University of Miami, Miami, FL 33101, USA; ihentall@med.miami.edu

**Keywords:** spinal cord injury, spinal cord stimulation, deep brain stimulation, neuromodulation

## Abstract

Traumatic spinal cord injury (SCI) is a devastating neurological condition characterized by a constellation of symptoms including paralysis, paraesthesia, pain, cardiovascular, bladder, bowel and sexual dysfunction. Current treatment for SCI involves acute resuscitation, aggressive rehabilitation and symptomatic treatment for complications. Despite the progress in scientific understanding, regenerative therapies are lacking. In this review, we outline the current state and future potential of invasive and non-invasive neuromodulation strategies including deep brain stimulation (DBS), spinal cord stimulation (SCS), motor cortex stimulation (MCS), transcutaneous direct current stimulation (tDCS) and repetitive transcranial magnetic stimulation (rTMS) in the context of SCI. We consider the ability of these therapies to address pain, sensorimotor symptoms and autonomic dysregulation associated with SCI. In addition to the potential to make important contributions to SCI treatment, neuromodulation has the added ability to contribute to our understanding of spinal cord neurobiology and the pathophysiology of SCI.

## 1. Introduction

Traumatic spinal cord injury (SCI) is a devastating neurological disorder with a reported incidence in various countries and regions ranging from 10 to 80 million per population per year [1,2,3]. It is most commonly caused by road traffic accidents, falls, violence and sports injuries. The incidence and distribution of causes, and long-term survival rates, vary significantly across the globe, depending upon a wide range of complex social and economic factors [1,3,4]. It has a 2.5–5 fold higher incidence in males, with a peak in young adults (age 20–30) [1].

SCI is characterized by a constellation of symptoms including paralysis, paraesthesia, pain, cardiovascular, bladder, bowel and sexual dysfunction. The level of physical disability depends on the severity of the injury and the level of injury. The severity of the injury is commonly assessed by the International Standards for Neurological Classification of Spinal Cord Injury (ISCNSCI) developed by the American Spinal Injury Association (ASIA). This can be classified into different grades on the ASIA impairment scale (Table 1). Epidemiological studies suggest that more injuries occur at the cervical levels than thoracic levels, although the exact distribution varies based on the geographic location of the studies [1]. SCI has been shown to cause significant autonomic dysfunction, with neurogenic shock one of the leading causes of death [5].

Arguably at least as important as the physical and physiological disability caused by SCI are its psychological and economic impacts. SCI predominantly affects a young adult population and the psychological impact of rendering an independent, healthy individual paraplegic or tetraplegic without bladder, bowel or sexual function can be devastating. In addition to the costs associated with medical care in SCI patients, there is an immense secondary economic burden associated with individuals incapacitated for most of their prospective career and the subsequent impact upon their families. One study estimated the lifetime economic burden of 1.5 million 2011 Canadian dollars per tetraplegic individual [6].

In this review, we briefly outline the current management of acute and chronic SCI and focus on the potential of neuromodulation strategies in its treatment. In particular, we review the available clinical evidence for strategies such as deep brain stimulation (DBS), spinal cord stimulation (SCS), motor cortex stimulation (MCS), transcutaneous direct current stimulation (tDCS) and repetitive transcranial magnetic stimulation (rTMS) in affecting pain, sensorimotor symptoms and autonomic dysregulation, all of which are important sequelae in SCI.

## 2. Current Management of Acute Spinal Cord Injury

The mainstay of current management of acute spinal cord injury involves acute resuscitation and the prevention of secondary injury to the spinal cord. Acute resuscitation follows Advanced Trauma Life Support principles with particular importance to the airway and breathing in high cervical injuries that can impair diaphragmatic function, along with early spinal immobilisation. Circulatory resuscitation is also of paramount importance due to the phenomenon of neurogenic shock, which can cause profound bradycardia, hypotension and vasodilatation due to the loss of sympathetic tone. Standard first procedures include acute stabilisation, clinical assessment (ASIA score) and computerised tomography and magnetic resonance imaging (CT and MRI). Secondary injury to the spinal cord may be prevented by maintaining adequate perfusion to the cord [7,8] and early decompressive surgery [9,10,11]. There is also a growing body of evidence supporting intra-spinal cord pressure monitoring and expansion duraplasty for refractory intra-spinal hypertension, drawing parallels from intracranial pressure monitoring paradigms in traumatic brain injury [12,13,14,15].

There are currently no generally approved medical therapies to improve outcomes in acute SCI [8], although a number of major trials are ongoing. Riluzole, a blocker of voltage-gated sodium channels, has shown promise in improving motor function following cervical injury and is currently the subject of a phase III randomised controlled trial [16,17,18]. A substance of long-term interest has been the voltage-gated potassium channel blocker 4-aminopyridine [19], but this has given mostly disappointing results in acute and chronic human trials examining motor improvement [20,21,22]. Likewise, steroid treatment (methyl-prednisolone), which some practitioners viewed favourably in the previous two decades, now appears to offer little benefit and non-negligible risk [8,23]. Induced hypothermia remains a moderately promising therapy for acute SCI [24]. This was explored over many decades to treat early traumatic brain injury, although with variable results. Unfortunately, its critical dependence on both the timing of cooling post-injury and the level of temperature drop appear to be largely responsible for its variable efficacy in central nervous system (CNS ) neurotrauma [25,26]. Hypothermia is a typical acute sequela of spinal cord trauma, and is proposed to be an input to brainstem centers controlling repair, as described below.

Subsequent management involves a combination of intense rehabilitation to optimise functional outcomes and medical therapies, designed either to prevent complications, such as venous thromboembolic events, infections associated with indwelling catheters and pressure ulcers and osteoporosis or to treat specific symptoms such as neuropathic pain.

## 3. Current Management of Chronic Spinal Cord Injury

Despite the advances of modern molecular and cellular science, there has been little clinical progress in regenerative and restorative therapies for chronic SCI. In vitro and in vivo models have often looked promising, but no disease modifying therapies have been successfully translated to humans [27]. Following SCI, structurally damaged axons are unable to regenerate due to a combination of a glial inhibitory environment and a fundamental lack of neuronal intrinsic regeneration potential. These factors contribute to persisting functional neurological impairment. A number of promising restorative and regenerative therapies, including the delivery of olfactory ensheathing cells to act as a scaffold [28,29], chondroitinase therapy to limit the glial scar [30] and taxol therapy to promote microtubule stabilization [31], have sought to target these mechanisms, with limited clinical success [27].

The various proposals for using CNS stimulation in SCI and other neurotrauma are in part prompted by the apparent obstacles to other approaches. Electrical stimulation of a discrete anatomical pathway elicits a natural response that can be helpful, whereas the ultimate benefits of administering a mixture of slow-acting, powerful drugs with an array of adverse effects and issues of receptor specificity, timing of actions, dosing regimen, tolerance and spatial targeting remain unclear. Neurotrophic substances of potential interest additionally run the risk of uncontrolled deleterious axonal sprouting [32]. The testing of restorative pharmacotherapies to satisfy scientific standards and regulatory demands can also present major difficulties. Cell transplantation may be free of some of these problems, but safe functional integration of exogenous cells within solid tissue is also a formidable task, and there is the potential for tumor formation with some cell types [33,34]. The routine clinical use of cell transplantation therefore seems to be well beyond the immediate horizon.

## 4. The Potential Roles for Neurostimulation in SCI

The indications for functional neurosurgery, including deep brain stimulation (DBS), cerebral cortical stimulation and spinal cord stimulation (SCS), have expanded drastically over the last decade and now encompass a whole host of neurological disorders including movement disorders, psychological and psychiatric disorders, pain (including neuropathic pain and primary headache disorders), epilepsy, disorders of consciousness and cognitive disorders [35,36].

In the context of SCI, functional procedures have the potential to impact upon a number of different domains, including pain, functional motor/sensory recovery, bladder/bowel function and cardiovascular autonomic dysregulation. Many of these have been highlighted as important symptoms to address in large surveys of SCI patients [37,38].

**Figure 1:**
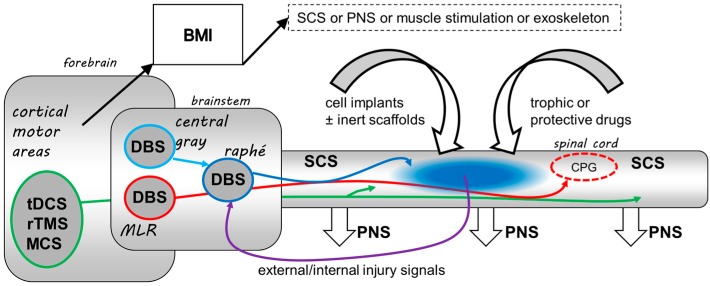
Diagram of possible sites for therapeutic electrical stimulation and other common interventions in spinal cord injury (SCI). Deep brain stimulation (DBS) of a brainstem restorative feedback loop is proposed to augment restorative effects around the injury site. This treatment resembles cell implantation or drug treatments in that it aims for non-specific recovery of visceral and sensory-motor deficits. Most forms of stimulation are concerned with narrowly specified functions. Thus DBS in brainstem central grey may also be used to block neuropathic pain. The nearby mesencephalic locomotor region (MLR) can be stimulated to activate descending pathways that boost the locomotor central pattern generator (CPG) in lower thoracic and upper lumbar segments. Cortical stimulation activating corticospinal tracts, whether non-invasively via transcutaneous direct current stimulation (tDCS) or repetitive transcranial magnetic stimulation (rTMS), or invasively with direct motor cortex stimulation (MCS), can be used for immediate production of movement or to induce adaptive plastic changes in motor output. Cortical commands may also be fed to a brain-machine interface (BMI) to control variously situated electrodes for peripheral nerve stimulation (PNS) or spinal cord stimulation (SCS). SCS distal to the injury has been used for bladder control; proximal SCS can be used block pain; and stimulation at any spinal location can be used to generate movements, depending on the degree of completeness of functional loss. The simplicity and comprehensiveness of restorative DBS in the brainstem are points in its favour.

There are three broad strategies for chronic SCI: (i) using technology to restore function without restoring neural architecture; (ii) taking advantage of plasticity to harness residual circuitry; and (iii) encouraging active regeneration of injured neurons. These are depicted in Figure 1.

The first strategy involves suppressing or inducing immediate effects. Pain suppression is an obvious example, and the most clinically developed (see next section). Less obviously, one can try to evoke or improve some specific movements that have been lost. For example, to facilitate locomotor movements in incompletely injured individuals, the mesencephalic locomotor region can be stimulated, thus overcoming weakness in the spinal central pattern generator [39]. This positive approach requires in practice some integration with patient input or feedback so that it is turned on only when safe and needed. A more complex way to provide control signals to stimulators is by brain-machine interfaces tapping neuronal firing in neocortex that represents motor commands [40]. This has been successfully implemented via wireless control connecting cortical microelectrodes to lumbar epidural stimulators in monkeys [41]. However, a key obstacle that must be understood and overcome is the so-called “foreign body response” that reduces the efficacy of implants over time [42].

The second strategy focuses on stimulation of pathways assumed to possess considerable neuroplasticity in their normal functioning, pre-eminently the cortico-spinal tract [43]. This propensity for axonal growth and sprouting can perhaps be exploited by the application of stimulation, but it is unclear how exactly to use this to shape a valid, adaptive repair.

A final strategy that has met with some preclinical success assumes that certain brain regions are specifically adapted as centres for repairing recent mild neurotrauma [44,45,46,47]. Thus the serotonergic raphe nuclei of the brainstem release a cocktail of trophic substances from their ubiquitous axon terminals in response to injury-correlated sensory or chemical stimuli (e.g., nociception, unconsciousness, hypothermia, circulating cytokines). Stimulation of this feedback loop, or the regions that feed into it, such as the midbrain periaqueductal grey (PAG), have been shown to enhance early histological and sensory-motor recovery in rats with incomplete thoracic SCI [35,36,37]. A potential advantage of these brain centres is that they are non-eloquent, that is, the applied stimulus intensities evoked no observable motor responses or arousal changes, facilitating continuous long-term stimulation.

## 5. Neurostimulation for Pain Following SCI

Pain is defined by the International Association for the Study of Pain (IASP) as “an unpleasant sensory and emotional experience associated with actual or potential tissue damage, or described in terms of such damage” [48]. In the context of SCI, pain is common, affecting over 70% of SCI patients, and can be categorised as neuropathic or nociceptive (musculoskeletal) in origin, affecting 34% and 64% of long-term survivors respectively [49,50,51,52,53,54,55]. Pain following SCI is thought to occur due a combination of abnormal inputs from the injured spinal cord and aberrant reorganisation of cortical circuitry [56,57] and has been shown to be more common at one year following injury than immediately after [55]. The multifactorial aetiology of SCI-related pain makes it notoriously difficult to treat, especially with traditional pharmacotherapy [58,59] and the IASP’s Neuropathic Pain Special Interest Group is unable to currently recommend any interventional strategies for the management of SCI-related pain [60].

The use of neurostimulation to treat pain was reported as early as 1960 [61], predating the Melzack & Wall gate theory [62]. Current indications of neurostimulation for pain include pain from failed back surgery syndrome (FBSS), neuropathic pain secondary to peripheral nerve injury (e.g., amputation, brachial plexus injury), facial pain and headache disorders [63,64]. Interventions can target any part of an impaired nociceptive pathway, including at the level of the spinal cord, deep brain nuclei or motor cortex [65,66].

Evidence for the efficacy of DBS in the context of SCI-related pain is limited to a few patients that form part of small series which have had varying results [63,64,67,68,69,70,71,72,73]. The main sites targeted have been ventralis posterior lateralis (VPL) nucleus of the thalamus and the midbrain central gray, comprising the periaqueductal grey (PAG) and periventricular grey (PVG). A systematic review by Previnaire in 2009 recorded 19 cases of the successful implantation of DBS electrodes in SCI patients following trial stimulation in 36 patients to the VPL ± central gray. Only three (16%) of these cases reported “long-term success” [69]. DBS of VPL may also have a role in the management of phantom sensations and pain following SCI [74]. More recently, DBS electrodes in the central gray in two SCI patients were optimised to provide best analgesia; this study found that very low frequency stimulation (effectively <0.67 Hz) was most effective in these two patients; added benefits included a reduction in side effects and a long battery life and the authors emphasized the importance of allowing time (hours to days) before assessing the efficacy of new stimulation parameters [75]. Improving knowledge of the somatotopic organisation of the PAG may aid optimal electrode targeting, and given the bilateral pain often experienced by SCI patients, it is likely that most SCI patients will need bilateral electrodes [76,77]. Based on evidence of the success on cingulotomy for cancer related pain, Spooner et al. reported a single case of effective analgesia in SCI following DBS to the rostral anterior cingulate cortex, although this patient was only followed up for four months [78]. A subsequent case series of patients treated with anterior cingulate cortex DBS for chronic pain of various aetiologies (including 1 with SCI) illustrates its potential for long-term control [79]. Over the years, the lack of randomised controlled clinical trial evidence has led to a decrease in the number of studies reporting on DBS for pain [57] and this has been confounded by the US Food and Drug Administration giving DBS for pain “off label” status [80], so that the International Neuromodulation Society still views intracranial modulation for pain as “investigational” [81].

Another option for SCI-related pain is epidural motor cortex stimulation (MCS), although the limited reports in the literature suggest that SCI-related pain responds poorly to MCS compared with other pain syndromes [68,82]. However, the systematic review by Previnaire concluded that MCS may be more effective than DBS in the context of SCI, providing “long-term success” in four out of seven patients [69].

SCS has been used to treat a variety of pain syndromes for over 40 years and the first reports of its use for spinal cord injury date back to 1972 [83]. It is based upon the Melzack and Wall gate theory that stimulation of the large dorsal column fibres will inhibit some of the activity produced by smaller myelinated and unmyelinated fibres in the dorsal horn [62]. Nashold’s series of 30 patients includes five with traumatic SCI (three from spinal fractures, one with a gunshot wound and one with a cord contusion), none of whom had an “excellent” response to the treatment [83]. Since then, reports of the utility of SCS for SCI-related pain have been limited and suggest poorer responses in SCI patients compared to other indications such as failed back surgery and peripheral neuropathy pain [84,85,86]. This may be explained by injury to the neural circuits underlying its efficacy but we are still far from understanding the mechanisms underlying the efficacy and failure of SCS, especially in the context of SCI. Newer modalities of SCS such as burst and high frequency stimulation are yet to be evaluated in their efficacy for chronic pain after SCI.

Non-invasive neuromodulatory strategies include repetitive transcranial magnetic stimulation (rTMS) and transcranial direct current stimulation (tDCS), both of which are purported to work on the basis of altering maladaptive plasticity within pain circuits, affecting nuclei in the thalamus and subthalamic regions [59,87,88]. A recent systematic review identified six studies assessing 127 patients with neuropathic pain treated with rTMS following SCI. It concluded that although there was some reduction in pain indices following rTMS, this did not reach statistical significance [89]. There are many unresolved controversies within the field of rTMS including the location (motor cortex versus premotor cortex/dorsolateral prefrontal cortex), type and orientation of coil, schedule of repetitive stimulation and persistence of therapeutic response [90,91,92,93]. However, given the non-invasive nature of rTMS, it could prove a useful tool both in terms of mechanistic understanding and therapeutic benefit for pain following SCI. It may have a role in predicting responsiveness to MCS and the ability to influence phantom sensations after SCI, which could provide significant functional benefits for these patients [94,95]. tDCS is less well characterised, and differs from rTMS in that it does not result in neuronal firing but changes the resting membrane potential, thereby altering neuronal excitability. It has long-term effects that are thought to be caused by altered neurotransmitter systems [96]. The largest clinical study to date utilised the anode over the primary motor cortex and showed a reduction in pain VAS scores in 16 patients following SCI. The tDCS was found to alter metabolism in the subgenual anterior cingulate cortex, left dorsolateral prefrontal cortex and insula, suggesting an effect of tDCS on the emotional and cognitive components of pain [97]. The evidence in support of tDCS has led to it being included as part of third-line therapies for neuropathic pain for SCI in the CanPain guidelines; it is the only neuromodulatory strategy to be included in the guidelines [98].

The future of neurostimulation for pain in SCI lies in improving the level of evidence for each intervention and identifying subsets of patients and types of pain that benefit from specific interventions. Presently, DBS for various types of pain can yield a range of effects in different patients from dramatic and highly pleasing success to disheartening failure, and the factors leading to these variable outcomes are entirely unclear, although there are some weak statistical predictors [77]. This task will no doubt be aided by homogenisation of the measurement of pre-intervention and post-intervention data sets, such as the International Spinal Cord Injury Pain Basic and Extended Data Sets [99,100]. In addition to the rigorous assessment of outcomes, surgical complications must also be taken into consideration; studies have highlighted the burden of complications associated with implanted stimulators, which can impact upon quality of life and overall outcomes [69].

## 6. Neurostimulation for Sensorimotor Recovery Following SCI

There is little in the way of clinical evidence for the efficacy of neuromodulation for locomotor and sensory recovery following spinal cord injury. Perhaps the best evidence for efficacy of DBS in animal models comes from two landmark studies, showing the efficacy of DBS in improving gait in cord injured rats. The two studies used different targets, the mesencephalic locomotor region (MLR) and the nucleus raphe magnus and the PAG [39,47], and deliver improvement in different time frames (immediate or long-term). The correlates of this circuitry in humans need to be better understood, especially since the efficacy of stimulation for motor responses depends on the number of residual fibres within the injured cord [101].

Similar rodent and mammalian evidence also exists for SCS, which has been improved by the use of closed-loop feedback systems to refine stimulation parameters and improve gait [102,103,104]. The putative role for SCS can also be extrapolated from studies observing motor benefit in patients and animals with Parkinson’s disease [105,106] and multiple sclerosis [107,108] undergoing SCS. There is preliminary human evidence to suggest that epidural SCS may be efficacious in producing electrophysiological improvements in patients with SCI [109,110,111]. Further work is necessary to translate these electrophysiological improvements into functional benefits. Developing a better understanding of the pattern generator for human gait may also indicate a use for lumbosacral epidural stimulation, obviating the need for supraspinal inputs [112]. This concept of using stimulation to replace lost supraspinal input has recently been demonstrated in a primate model, which relies on real-time input of cortical signals to optimise lumbar epidural stimulation [41,113]. However, this model is limited by the lack of a sensory feedback system, which would further optimise motor functional recovery.

Animal evidence also exists for the utility of combined MCS and spinal tDCS in promoting motor recovery after pyramidotomy, suggesting that stimulation proximal and distal to the injury could facilitate the improvement in function following SCI [114]. TMS may also have a role in motor recovery, as it has been shown to elicit EMG responses below the level of injury in individuals with motor-complete SCI; it may therefore have a role in identifying individuals who may benefit from neuromodulatory therapies or have a therapeutic role in its own right [115,116].

## 7. Neurostimulation for Autonomic Recovery Following SCI

Autonomic functions, including bladder, bowel and sexual function, have consistently been highlighted as priorities for recovery in patients with SCI [37,38]. In the acute phase, bladder dysfunction often manifests as a “flaccid paralysis” resulting in urinary retention requiring catheterisation. In the chronic phase, this progresses into detrusor hyperreflexia and detrusor-sphincter dyssynergia resulting in incontinence and incomplete voiding; the mainstay of current treatment is intravesical botulinum toxin injection which requires repeated treatments every few months and does not address the dyssynergia [117,118]. Sexual dysfunction can manifest in a number of different ways in males and females, depending upon the level and completeness of the injury [117]. In addition to the physical impact, sexual dysfunction in particular may impact psychological wellbeing and overall quality of life [119,120]. Gastrointestinal disturbances in SCI are common (>60%) and wide-ranging, including delayed gastric emptying, abnormal colonic myoenteric activity and sphincter/defecation dysfunction [117,121,122]. The lower GI disturbances are consistent with a “spastic” paresis of the bowel. The increased colonic muscle tone, abnormal rectal compliance and tight external sphincters require laxatives and digital reflex stimulation to promote bowel emptying [117,123].

Cardiovascular autonomic dysfunction is a major problem in SCI, and contributes to the excess cardiovascular mortality in SCI [124]. In the acute phase, the loss of sympathetic outflow can lead to devastating hypotension, hypothermia and dysrhythmias [125]. In the chronic phase, especially after cervical or high thoracic injuries, disturbances such as orthostatic hypotension and autonomic dysreflexia (sudden drastic episodes of elevated blood pressure and bradycardia) have been shown to be common in SCI patients, which may contribute to the increased cardiovascular disease risk and also affect other aspects of recovery such as rehabilitation and cognitive function [5,125,126,127,128]. SCI can also lead to thermoregulatory dysfunction.

Although there is little in the way of evidence for autonomic manipulation in the context of SCI, evidence from other conditions indicate that neuromodulation strategies may have a role [129]. There is evidence that DBS can affect urinary symptoms and control in Parkinson’s disease, dystonia, essential tremor and chronic pain [130,131,132,133]. These studies show a predominant effect on lower urinary tract symptoms and mechanistic studies have shown that this might be due to enhanced processing of bladder information following subthalamic nucleus DBS. In the context of SCI, this could prove beneficial in patients with incomplete SCI, where enhancement of residual sensory inputs could improve continence. In one study where patients had PAG stimulators for chronic pain, the maximum cystometric capacity increased during saline infusion into the bladder when the stimulators were switched on, indicating a switch to the “filling” state over the “voiding” state [134]. However, a deeper understanding of the subcortical networks involved in urinary continence in health and how they are disturbed in SCI are required. A single patient has also been reported in the literature to have improved bowel symptoms following subthalamic nucleus DBS for Parkinson’s, which may provide benefit via a similar mechanism [135].

DBS has also been shown to affect blood pressure with dorsal central gray stimulation resulting in an increase in blood pressure and ventral central gray stimulation resulting in a decrease in blood pressure [136,137,138,139,140,141]. There are therefore potential avenues to treat both the orthostatic hyptension and autonomic dysreflexia seen in SCI patients. Ventral versus dorsal DBS in the central gay has also been shown to alter different frequency components of heart rate variability, suggesting differential contributions of parasympathetic and sympathetic nervous systems to its mechanisms of action [142].

A number of other neuromodulatory strategies have also been reported for autonomic recovery, specifically for bladder, bowel and sexual function [118]. Although the sacral anterior root stimulator (Brindley device) has been shown to be successful at overcoming detrusor-sphincter dyssynergia and achieving and sustaining continence in 88% of 500 patients at a mean of four years follow-up [143], the negative impacts of deafferentation of the posterior nerve roots on bladder, bowel and sexual function has seen its use decline [118]. More recent evidence of using the same device for anterior and posterior root stimulation has shown promise [144,145]. Sievert and colleagues present compelling data that early (median 2.9 months following injury) insertion of sacral nerve modulators into the S3 nerve roots can improve urinary continence, bowel function and even help achieve erection in patients with complete SCI [144].

## 8. The Future of Neurostimulation in SCI

The plethora of applications for neuromodulation in SCI provides promise in a field currently devoid of disease-modifying therapies. The physiological, psychological and economic benefits associated with addressing issues such as pain, mobility and autonomic symptoms in SCI patients is large. Given the current dearth of treatment options, neuromodulation is an attractive emerging option. However, there are a number of outstanding questions that first need to be answered.

Apart from the need to identify evidence-based indications for deep brain, motor cortex and spinal cord stimulation and the non-invasive strategies in the context of SCI, we also need a more nuanced understanding of the patient, injury and treatment factors that influence success. Specifically, the timing of interventions seems a crucial variable. The interventions for pain have thus far been performed many months to years after the injury once aberrant circuitry has become established and it remains to be explored whether more acute interventions could be more beneficial at preventing the development of maladapted circuits or promote regeneration [146]. Another key variable are the stimulation parameters. As recent studies have shown, parameters that are vastly different to what is commonly used may be more beneficial [75] and optimisation of parameters may be aided by closed-loop feedback systems, as has been tentatively shown in Parkinson’s disease [147,148].

The potential, in particular, of PAG DBS for addressing multiple symptoms is intriguing. The animal and human evidence presented earlier in this review have illustrated the potential to address pain, autonomic features and facilitate motor recovery in SCI. The fact that PAG is already a target in human DBS for pain makes it an ideal candidate for further investigation to target the disabling triad of pain, autonomic dysreflexia (both cardiovascular and sphincteric) and paralysis.

Mechanistic insight studies, both in humans and animals, will also aid progress in the field. In particular, human electrophysiological and imaging studies in spinal cord injury both before and after neurostimulator implantation will allow us to develop a better understanding the biological processes underlying symptomatic improvement. For example, despite the use of DBS in the central gray for pain, the mechanism of action remains unclear [149]. Theories implicating endorphins and opioid pathways have since been disproven [150,151,152]. More recent evidence has shown that DBS and SCS may modulate gene expression [153,154]. An understanding of these modifications may provide insight into the failure of CNS regeneration following injury. However, as with other SCI studies, translation of mechanistic understanding in animals to humans must be approached with caution [27].

Technological advances will also be of direct relevance to this field. Smaller nanodevices for stimulation may improve the specificity and reduce the side-effect profile. Such devices have also been reported to promote axonal regeneration in the cord [155] and may allow access to previously inaccessible nuclei, such as the nucleus raphe magnus that has been shown to improve gait in rats [47]. Crucial to providing conclusive evidence of efficacy is well designed clinical trials with relevant outcome measures that are able to be consistently replicated. Much is to be learnt from the failure of medical therapies such as steroids [156]. Trials in SCI will never be able to recruit large numbers, which places added importance on trial design. An abundance of prognostic factors (ASIA score on admission, age, level of injury, rehabilitation) increases heterogeneity in SCI trials. Adaptive trial designs or n-of-1 trials [157] may be important in the context of neuromodulation, as will be improving our understanding of factors influencing outcome in SCI patients. Using established frameworks such as the IDEAL recommendations and using homogenised core outcome sets will be important to systematic evaluation of these new technologies [158,159].

Despite the potential of neuromodulation strategies, the limitations must also be taken into consideration. All implants and surgical procedures are associated complications such as the foreign body response that reduces efficacy over time [42]. Specifically, the risks of functional compromise associated with damage to residual neural structures in SCI patients may be higher than in other disorders.

## 9. Conclusions

Despite the current paucity of clinical evidence for efficacy, functional neurosurgery has the potential to make contributions to the treatment of SCI. It can immediately relieve some of the many visceral and sensory-motor deficits and has the potential to effectuate some useful degree of reversal of the underlying neurodegeneration, albeit partially, by exploiting the capacity of electrical activity to increase sprouting or induce other plastic changes in neural pathways, such as maturation and integration of endogenous neural progenitor cells.

## Figures and Tables

**Table 1:** The American Spinal Injury Association (ASIA) Impairment Scale.

ASIA Impairment Scale	Definition	Explanation
A	Complete	No motor or sensory function is preserved in the sacral segments S4–S5.
B	Incomplete	Sensory but not motor function is preserved below the neurological level and includes the sacral segments S4–S5.
C	Incomplete	Motor function is preserved below the neurological level, and more than half of the key muscles below the neurological level have a muscle grade less than 3.
D	Incomplete	Motor function is preserved below the neurological level, and more than half of the key muscles below the neurological level have a muscle grade of 3 or more.
E	Normal	Motor and sensory function are normal.
